# Endogenous retroviral promoter exaptation in human cancer

**DOI:** 10.1186/s13100-016-0080-x

**Published:** 2016-12-01

**Authors:** Artem Babaian, Dixie L. Mager

**Affiliations:** 1 Terry Fox Laboratory, British Columbia Cancer Agency, 675 West 10th Avenue, Vancouver, BC V5Z1L3 Canada; 2 Department of Medical Genetics, University of British Columbia, Vancouver, BC Canada

**Keywords:** Gene regulation, Endogenous retrovirus, Long terminal repeat, Retrotransposon, Epigenetics, Cancer, Alternative promoter, Exaptation, Transcription

## Abstract

Cancer arises from a series of genetic and epigenetic changes, which result in abnormal expression or mutational activation of oncogenes, as well as suppression/inactivation of tumor suppressor genes. Aberrant expression of coding genes or long non-coding RNAs (lncRNAs) with oncogenic properties can be caused by translocations, gene amplifications, point mutations or other less characterized mechanisms. One such mechanism is the inappropriate usage of normally dormant, tissue-restricted or cryptic enhancers or promoters that serve to drive oncogenic gene expression. Dispersed across the human genome, endogenous retroviruses (ERVs) provide an enormous reservoir of autonomous gene regulatory modules, some of which have been co-opted by the host during evolution to play important roles in normal regulation of genes and gene networks. This review focuses on the “dark side” of such ERV regulatory capacity. Specifically, we discuss a growing number of examples of normally dormant or epigenetically repressed ERVs that have been harnessed to drive oncogenes in human cancer, a process we term onco-exaptation, and we propose potential mechanisms that may underlie this phenomenon.

## Background

Sequences derived from transposable elements (TEs) occupy at least half the human genome [1, 2]. TEs are generally classified into two categories; DNA transposons, which comprise 3.2% of the human genome; and the retroelements, short interspersed repeats (SINEs, 12.8% of the genome), long interspersed repeats (LINEs, 20.7%) and long terminal repeat (LTR) elements, derived from endogenous retroviruses (ERVs, 8.6%). Over evolutionary time, TE sequences in the genome can become functional units that confer a fitness advantage, a process called “exaptation” [3, 4]. Exaptation includes protein coding, non-coding and regulatory effects of TEs. This is in contrast to the designation of “nonaptations” for genetic units that perform some function (such as initiate transcription) but don’t impact host fitness [4]. Besides their roles in shaping genomes during evolution, TEs continue to have impact in humans through insertional mutagenesis, inducing rearrangements and affecting gene regulation, as discussed in recent reviews [5–12].

Efforts to explore the role of TEs in human cancer have focused primarily on LINEs and ERVs. While nearly all L1s, the major human LINE family, are defective, a few hundred retain the ability to retrotranspose [13] and these active elements occasionally cause germ line mutations [9, 14, 15]. Several recent studies have also documented somatic, cancer-specific L1 insertions [16–23], and a few such insertions were shown to contribute to malignancy [9]. For example, two L1 insertions were documented to disrupt the tumor suppressor gene *APC* in colon cancer [16, 23]. However, it is probable that most insertions are non-consequential “passenger mutations”, as recently discussed by Hancks and Kazazian [9]. Thus, the overall biological effect size of LINE retrotransposition on the process of oncogenesis may be limited.

No evidence for retrotranspositionally active ERVs in humans has been reported [24–26], so it is unlikely that human ERVs activate oncogenes or inactivate tumor suppressor genes by somatic retrotransposition. This is in contrast to the frequent oncogene activation by insertions of exogenous and endogenous retroviruses in chickens or mice, where retrotranspositional activity of ERVs is very high [27–29]. Therefore, to date, most studies into potential roles for ERVs in human cancer have focused on their protein products. Indeed, there is strong evidence that the accessary proteins Np9 and Rec, encoded by members of the relatively young HERV-K (HML-2) group, have oncogenic properties, particularly in germ cell tumors [30–33].

Regardless of their retrotranspositional or coding capacity, ERVs may play a broader role in oncogenesis involving their intrinsic regulatory capacity. De-repression/activation of cryptic (or normally dormant) promoters to drive ectopic expression is one mechanism that can lead to oncogenic effects [34–40]. Because TEs, and especially ERV LTRs, are an abundant reservoir of natural promoters in the human genome [6, 41, 42], inappropriate transcriptional activation of typically repressed LTRs may contribute to oncogenesis. Here we review examples of such phenomena, which we term “onco-exaptation”, and propose two explanatory models to understand the role of LTRs in oncogenesis.

### Promoter potential of ERVs

Hundreds of ERV “families” or groups, which is the more proper designation [43], are remnants of ancient retroviral infections of the germ line and occupy at least 8.67% of the human genome [1, 24, 44]. These range from groups that integrated before the divergence of rodents and primates, such as older members of the large MaLR/ERV-L class, to the youngest HERV-K (HML-2) group, a few members of which are insertionally polymorphic in humans [45, 46]. While it has been postulated that rare “active” HERV-K elements exist at very low allele frequencies [45], there is currently no evidence for new somatic or germ line insertions of ERVs in humans and nearly all have lost coding potential [24–26]. The situation is starkly different in inbred mice, where at least 10% of documented, phenotype-producing germ line mutations and numerous somatic, cancer-associated insertions are due to ongoing retrotranspositions of ERVs [28, 29, 47]. Table 1 lists select major ERV groups found in humans, members of which are mentioned in this review.

**Table 1:** ERV/LTR groups mentioned in this review

ERV Class	ERV Group	Associated LTRs (Repbase names)	~Copies of internal regions^a^	~Copies of solitary LTRs^b^
I (ERV1)	HERV-H	LTR7, 7B, 7C, 7Y	1060	1270
	HERV-9	LTR12, 12B-12 F	450	6500
	HERV-E	LTR2, 2B, 2C	250	720
	HUERS-P2	LTR1, 1A-1 F	120	3000
	LOR1	LOR1a, 1b	175	1080
	MER41	MER41A-41G	275	4110
II (ERVK)	HERV-K (HML-2)	LTR5, 5A, 5B, 5Hs	80	1200
III (ERVL)	HERV16	LTR16A-16E	860	18100
III (MaLR)	THE	THE1A-1D	7900	21260
	MLT1	MLT1A-1O	3820	146,550

^a^Copy numbers of internal regions estimated from Dfam (dfam.org) [50]

^b^Solitary LTR numbers estimated from Dfam coverage minus 2x internal region numbers, assuming all internal regions are associated with two LTRs

Approximately 90% of the “ERV-related” human genomic DNA is in the form of solitary LTRs, which are created over evolutionary time via recombination between the 5’ and 3’ LTRs of an integrated provirus [48, 49]. LTRs naturally contain transcriptional promoters and enhancers, and often splice donor sites, required for autonomous expression of the integrated LTR element. Furthermore, unlike for LINEs (see below), the integration process nearly always retains the primary transcriptional regulatory motifs, i.e. the LTR, even after recombination between the LTRs of a full-length proviral form. Mutations will degrade LTR promoter/enhancer motifs over time, but many of the >470,000 ERV/LTR loci in the genome [50] likely still retain some degree of their ancestral promoter/enhancer function, and hence a gene regulatory capacity.

LTR-mediated regulation of single genes and gene networks has been increasingly documented in the literature. For example, studies have implicated ERV LTRs in species-specific regulatory networks in ES cells [51], in the interferon response [52], in p53-mediated regulation [53], as tissue-specific enhancers [54, 55] and in regulating pluripotency by promoting genes and lncRNAs in stem cells [56–60]. LTR regulatory capacity arises from both their “ready-to-use” ancestral transcriptional factor (TF) binding sites and by mutation/evolution of novel sites, possibly maintained through epistatic capture [61] (recently reviewed in [42]). For more in depth discussion of the evolutionary exaptation of enhancers/promoters of LTRs and other TEs in mammals, we refer the reader to a rapidly growing number of reviews on this subject [6, 10, 42, 62–65]. Suffice it to say that, retrotranspositionally incompetent ERV LTRs, long considered the “poor cousin” of active L1 elements, have emerged from the shadowy realm of junk DNA and are now recognized as a major source of gene regulatory evolution through exaptation of their promoters and enhancers.

### Promoter potential of LINEs and other non-LTR TEs

Besides via new retrotransposition events, existing L1 elements can also impact genes through promoter donation. Full-length L1 elements harbor two internal promoters at their 5’ end, a sense promoter that drives expression of the element and an antisense promoter that has been shown to control expression of nearby genes through formation of chimeric transcripts [66–69]. Recently, this antisense promoter was also shown to promote expression of a small protein ORF0, which plays a regulatory role in retrotransposition [70]. While there are approximately 500,000 L1 loci in the human genome [1], the vast majority of them are 5’ truncated due to incomplete reverse transcription during the retrotransposition process. Only ~3500-7000 are full length, retaining their promoters and hence, the potential ability to lend these promoters to nearby genes [71, 72]. Therefore, irrespective of differences in promoter strength, epigenetic regulation or mutational degradation, the vast copy number difference (~500,000 LTRs versus ~5000 promoter-containing L1s), is likely a major reason why the great majority of TE-initiated transcripts involve LTRs rather than L1s. In genome-wide screens of TE-initiated transcripts, small fragments of old L2 elements, which do not span the canonical L2 promoter, can be found as TSSs of lowly expressed transcripts [73] (unpublished data). Such instances likely represent “*de novo”* promoters, those arising naturally from genomic DNA which happens to be derived from a TE fragment, (possibly because L2 fragments have a GC rich base composition), rather than an “ancestral” or “ready-made” promoter, one which utilizes a TE’s original regulatory sequence.

Human SINE elements, namely ALUs and the older MIRs, can also promote transcription of nearby genes but these instances are relatively rare [68] given their extremely high copy numbers (~1.85 million fragments) [50]. This likely partly reflects the fact that SINEs, being derived from small functional RNAs, inherently possess PolIII promoters, rather than PolII, and their autonomous promoter strength is weak [74, 75]. Old MIR elements, as well as other ancient SINEs and DNA TEs, have been more prominent as enhancers, rather than genic promoters, as shown in several studies [76–81].

### TEs and the cancer transcriptome

While some TE components have assumed cellular functions over evolutionary time, such as the *syncytin* genes in mammalian placenta, derived from independent ERV *env* genes in multiple mammals [6, 44, 82–84], the vast majority of TE/ERV insertions will be neutral or detrimental to the host. Given the potential for harm, multiple host mechanisms to repress these sequences have evolved. In mammals, ERV and L1 transcription is suppressed in normal cells by DNA methylation and/or histone modifications as well as many other host factors [9, 85–92]. The epigenetic regulation of TEs is relevant in cancer because epigenetic changes are common in malignancy and frequently associated with mutations in “epigenome-modifying” genes [93–97]. While the ultimate effects of many such mutations are not yet clear, their prominence indicates a central role for epigenomic dysregulation in oncogenesis [94, 98]. The most well established epigenetic changes are promoter hypermethylation and associated silencing of tumor suppressor genes [95, 99, 100] as well as genome-wide DNA hypomethylation [101–103]. Hypomethylation of ERVs and L1s in many tumors has been documented [104–106] and general transcriptional up-regulation of ERVs and L1s is often observed in cancers [33, 107–109]. However, other studies have shown no significant changes in ERV expression in selected human cancers compared to corresponding normal tissues [110, 111].

General conclusions about overall TE transcriptional deregulation in malignancy, or in any other biological state, are not always well founded and can depend on the type and sensitivity of the assay. For example, expression studies that use consensus probes for internal L1 or ERV regions to assay expression by custom microarrays or RT-PCR don’t resolve individual loci, so high expression signals could reflect dispersed transcriptional activation of many elements or the high expression of only one or a few loci. Such assays typically also cannot distinguish between expression due to TE promoter de-repression or due to increased transcription of transcripts harboring TEs. RNA-Seq has the potential to give information on expression of individual TE loci, but interpretations of expression levels can be confounded by mapping difficulties, length of read and sequencing depth [112]. In any event, in most cases where transcriptional up-regulation of TE groups or individual TEs has been detected in cancer, the biological relevance of such aberrant expression is poorly understood.

### Onco-exaptation of ERV/TE promoters

**Fig. 1:**
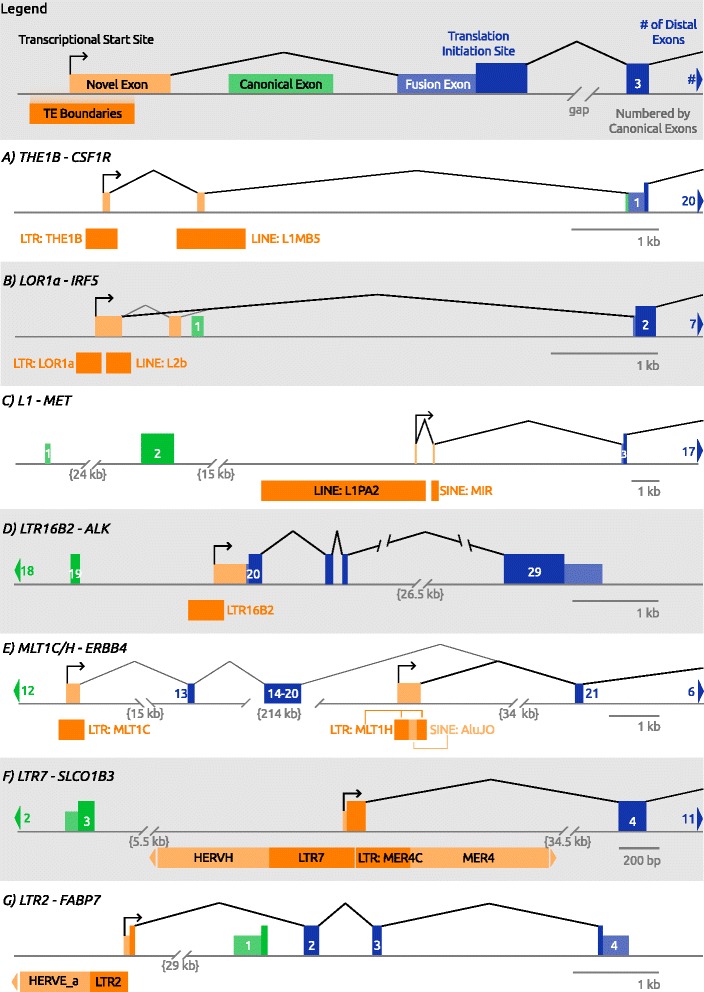
Examples of Onco-exaptation. Gene models of known TE-derived promoters expressing downstream oncogenes and listed in Table 2. Legend is shown at the top. **a** 6 kb upstream of *CSF1R*, a THE1B LTR initiates transcription and contains a splice donor site which joins to an exon within a LINE L1MB5 element and then into the first exon of *CSF1R*. The TE-initiated transcript has a different, longer 5’ UTR than the canonical transcript but the same full-length protein coding sequence. **b** An LOR1a LTR initiates transcription and splices into the canonical second exon of *IRF5* that contains the standard translational initiation site (TIS) to produce a full-length protein. There also is a novel second exon which is non-TE derived which is incorporated into a minor isoform of LOR1a-IRF5. **c** Within the canonical intron 2 of the proto-oncogene *MET*, a full length LINE L1PA2 element initiates transcription (anti-sense to itself), splicing through a short exon in a SINE MIR element and into the third exon of *MET*. The first TIS of the canonical *MET* transcript is 14 bp into exon 2, although an alternative TIS exists in exon 3, which is believed to also be used by the L1-promoterd isoform. **d** An LTR16B2 element in intron 19 of the *ALK* gene initiates transcription and transcribes into the canonical exon 20 of *ALK.* An in-frame TIS within the 20^th^ exon results in translation of a shortened oncogenic protein containing only the intra-cellular tyrosine kinase domain, but lacking the transmembrane and extracellular receptor domains of *ALK.* **e** There are two TE-promoted isoforms of *ERBB4,* the minor variant initiates in an MLT1C LTR in the 12^th^ intron and the major variant initiates in a MLT1H LTR in the 20^th^ intron. Both isoforms produce a truncated protein, although the exact translation start sites are not defined. **f** In the third exon of *SLCO1B3*, two adjacent partly full-length HERV elements conspire to create a novel first exon. Transcription initiates in the anti-sense orientation from an LTR7 and transcribes to a sense-oriented splice donor in an adjacent MER4C LTR, which then splices into the fourth exon of *SLCO1B3*, creating a smaller protein. **g** An LTR2 element initiates anti-sense transcription (relative to its own orientation) and splices into the native second exon of *FABP7*. The LTR-derived isoform has a non-TE TIS and splice donor which creates a different N-terminal protein sequence of FABP7

We propose that transcriptional up-regulation of LTR (and to a lesser extent L1) promoters is widespread in epigenetically perturbed cells such as cancer cells. Here we present specific published examples of onco-exaptation of TE-derived promoters affecting protein-coding genes (Table 2, Fig. 1). Although many other TE-initiated transcripts have been identified in cancer cells (see below), in this section we restrict the discussion to those cases where some role of the TE-driven gene in cancer or cell growth has been demonstrated.

**Table 2:** Activation of oncogenes by Onco-exaptation of TE-derived promoters

Gene^a^	Gene function	Primary result of TE-driven expression	TE type	TE promoter coordinates (hg38)	Cancer type	references
*CSF1R*	Tyrosine kinase receptor	Ectopic expression of normal protein	(ERVL-MaLR) THE1B LTR	chr5:150092453–150092809	HL	[113]
*IRF5*	Transcription factor	Ectopic expression of normal protein	(ERV1) LOR1a LTR	chr7:128936859–128937097	HL	[117, 119]
*MET*	Tyrosine kinase receptor	Protein truncation	(L1) L1PA2	chr7:116718498–116724489	CML, others?	[124, 125]
*ALK*	Tyrosine kinase receptor	Protein truncation	(ERVL) LTR16B2	chr2:29223783–29224196	melanoma	[38]
*ERRB4*	Tyrosine kinase receptor	Protein truncation	(ERVL-MaLR) MLT1C LTR (ERVL-MaLR) MLT1H2 LTR	chr2:211693702–211694209 chr2:211465146–211465419	ALCL	[129]
*SLCO1B3*	Anion transporter	Chimeric protein	(ERV1) LTR7^b^	chr12:20822187–20822617	colon, others	[133, 136]
*FABP7*	Fatty acid binding	Chimeric protein	(ERV1) LTR2	chr6:122748805–122749262	DLBCL	[138]

^a^Only those cases with supporting evidence of a role in the cancer are listed

^b^The fact that the promoter for these isoforms is an LTR was not noted in the cited papers

#### Ectopic and overexpression of protein-coding genes

The most straightforward interaction between a TE promoter and a gene is when a TE promoter is activated, initiates transcription, and transcribes a downstream gene without altering the open reading frame (ORF), thus serving as an alternative promoter. Since the TE promoter may be regulated differently than the native promoter, this can result in ectopic and/or overexpression of the gene, with oncogenic consequences.

The first case of such a phenomenon was discovered in the investigation of a potent oncogene colony stimulating factor one receptor (*CSF1R)* in Hodgkin Lymphoma (HL). Normally, *CSF1R* expression is restricted to macrophages in the myeloid lineage. To understand how this gene is expressed in HL, a B-cell derived cancer, Lamprecht et al. [113] performed 5’ RACE which revealed that the native, myeloid-restricted promoter is silent in HL cell lines, with *CSF1R* expression instead being driven by a solitary THE1B LTR, of the MaLR-ERVL class (Fig. 1a). THE1B LTRs are ancient, found in both Old and New World primates, and are highly abundant in the human genome, with a copy number of ~17,000 [50, 114] (Table 1). The *THE1B-CSF1R* transcript produces a full-length protein in HL, which is required for growth/survival of HL cell lines [113] and is clinically prognostic for poorer patient survival [115]. Ectopic *CSF1R* expression in HL appears to be completely dependent on the THE1B LTR, and CSF1R protein or mRNA is detected in 39–48% of HL patient samples [115, 116].

To detect additional cases of onco-exaptation, we screened whole transcriptomes (RNA-Seq libraries) from a set of HL cell lines as well as from normal human B cells for TE-initiated transcripts, specifically transcripts that were recurrent in HL and not present in normal B cells [117]. We identified the Interferon Regulatory Factor 5 gene (*IRF5*) as a recurrently up-regulated gene being promoted by a LOR1a LTR located upstream of the native/canonical TSS (Fig. 1b). LOR1a LTRs are much less abundant compared to THE1 LTRs (Table 1) but are of similar age, with the *IRF5* copy having inserted prior to New World-Old World primate divergence. *IRF5* has multiple promoters/TSSs and complex transcription [118] and, contrary to the *CSF1R* case, the native promoters are not completely silent in HL. However, LTR activity correlates with strong overexpression of the IRF5 protein and transcript, above normal physiological levels [117]. While our study was ongoing, Kreher et al. reported that *IRF5* is upregulated in HL and is a central regulator of the HL transcriptome [119]. Moreover, they found that IRF5 is crucial for HL cell survival. Intriguingly, we noted that insertion of the LOR1a LTR created an interferon regulatory factor-binding element (IRFE) that overlaps the 5’ end of the LTR. This IRFE was previously identified to be critical for promoter activity as a positive feedback loop through binding of various IRFs, including IRF5 itself [120]. Hence, the inherent promoter motifs of the LTR, coupled with the creation of the IRFE upon insertion, combined to provide an avenue for ectopic expression of IRF5 in HL.

#### Expression of truncated proteins

In these cases, a TE-initiated transcript results in the expression of a truncated open reading frame of the affected gene, typically because the TE is located in an intron, downstream of the canonical translational start site. The TE initiates transcription, but the final transcript structure depends on the position of downstream splice sites, and protein expression requires usage of a downstream ATG. Protein truncations can result in oncogenic effects due to loss of regulatory domains or through other mechanisms, with a classic example being *v-myb*, a truncated form of *myb* carried by acutely transforming animal retroviruses [121, 122].

The first such reported case involving a TE was identified in a screen of human ESTs to detect transcripts driven by the antisense promoter within L1 elements. Mätlik et al. identified an L1PA2 within the second intron of the proto-oncogene *MET* (*MET* proto-oncogene, receptor tyrosine kinase) that initiates a transcript by splicing into downstream *MET* exons (Fig. 1c) [67]. Not surprisingly, transcriptional activity of the CpG rich promoter of this L1 in bladder and colon cancer cell lines is inversely correlated to its degree of methylation [123, 124]. A slightly truncated MET protein is produced by the TE-initiated transcript and one study reported that L1-driven transcription of MET reduces overall MET protein levels and signaling, although by what mechanism is not clear [124]. Analyses of normal colon tissues and matched primary colon cancers and liver metastasis samples showed this L1 is progressively demethylated in the metastasis samples, which strongly correlates with increased L1-MET transcripts and protein levels [125]. Since MET levels are a negative prognostic indicator for colon cancer [126], these findings suggest an oncogenic role for L1-MET.

More recently, Wiesner et al. identified a novel isoform of the receptor tyrosine kinase (RTK), anaplastic lymphoma kinase (*ALK),* initiating from an alternative promoter in its 19^th^ intron [38]. This alternative transcription initiation (ATI) isoform or *ALK* ^ATI^ was reported to be specific to cancer samples and found in ~11% of skin cutaneous melanomas. *ALK* ^ATI^ transcripts produce three protein isoforms encoded by exons 20 to 29. These smaller isoforms exclude the extracellular domain of the protein but contain the catalytic intracellular tyrosine kinase domain. This same region of *ALK* is commonly found fused with a range of other genes via chromosomal translocations in lymphomas and a variety of solid tumors [127]. In the Wiesner et al. study it was found that ALK^ATI^ stimulates several oncogenic signaling pathways, drives cell proliferation in vitro, and promotes tumor formation in mice [38].

**Fig. 2:**
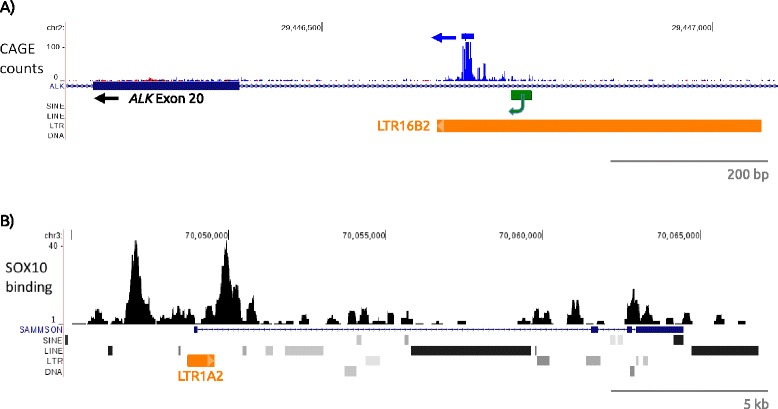
**a** UCSC Genome Browser view (hg19) of a portion of the human *ALK* gene. *ALK* exon 20 (large blue box) and a part of the upstream intron are shown, with direction of transcription from right to left. The LTR16B2 alternative promoter shown in the Repeatmasker track as an orange box and the 25 bp region of clustered TSSs in melanoma cells, identified using 5’ RACE by Weiser et al. [38], is shown as a green box The CAGE track above is from the Fantom5 project [128], with transcriptional direction indicated with a blue arrow. Most CAGE tags are from monocyte-derived macrophages and endothelial progenitor cells. **b** UCSC Genome Browser view (hg19) of the region encompassing the *SAMMSON* lncRNA, which plays an oncogenic role in melanoma [161]. The LTR1A2 promoter is indicated in the Repeatmasker track as an orange box. The ChIP-Seq track for SOX10 was created from a dataset (NCBI Gene Expression Omnibus: GSE61967) generated by Laurette et al. [225] in the 501Mel melanoma cell line

The *ALK* ^*ATI*^ promoter is a sense-oriented solitary LTR (termed LTR16B2) derived from the ancient ERVL family (Fig. 1d). LTR16B2 elements are found in several hundred copies in both primates and rodents [50, 114] and this particular element is present in the orthologous position in mouse. Therefore, the promoter potential of this LTR has been retained for at least 70 million years. Although not the first such case, the authors state that their findings “suggest a novel mechanism of oncogene activation in cancer through *de novo* alternative transcript initiation”. Evidence that this LTR is at least occasionally active in normal human cells comes from Capped Analysis of Gene Expression (CAGE) analysis through the FANTOM5 project [128]. A peak of CAGE tags from monocyte-derived macrophages and endothelial progenitor cells occurs within this LTR, 60 bp downstream of the TSS region identified by Wiesner et al. [38] (Fig. 2a), although a biological function, if any, of this isoform in normal cells is unknown.

To gain a molecular understanding of ALK-negative anaplastic large-cell lymphoma (ALCL) cases, Scarfo et al. conducted gene expression outlier analysis and identified high ectopic co-expression of *ERBB4* and *COL29A1* in 24% of such cases [129]. Erb-b2 receptor tyrosine kinase 4 (*ERBB4*), also termed HER4, is a member of the ERBB family of RTKs, which includes EGFR and HER2, and mutations in this gene have been implicated in some cancers [130]. Analysis of the ERRB4 transcripts expressed in these ALCL samples revealed two isoforms initiated from alternative promoters, one within intron 12 (I12-ERBB4) and one within intron 20 (I20-ERBB4), with little or no expression from the native/canonical promoter. Both isoforms produce truncated proteins that show oncogenic potential, either alone (I12 isoform) or in combination. Remarkably, both promoters are LTR elements of the ancient MaLR-ERVL class (Fig. 1e). Of note, Scarfo et al. reported that two thirds of ERBB4 positive cases showed a “Hodgkin-like” morphology, which is normally found in only 3% of ALCLs [129]. We therefore examined our previously published RNA-Seq data from 12 HL cell lines [117] and found evidence for transcription from the intron 20 MLTH2 LTR in two of these lines (unpublished observations), suggesting that truncated ERBB4 may play a role in some HLs.

#### TE-promoted expression of chimeric proteins

Perhaps the most fascinating examples of onco-exaptation involve generation of a novel “chimeric” ORF via usage of a TE promoter that fuses otherwise non-coding DNA to downstream gene exons. These cases involve both protein and transcriptional innovation and the resulting product can acquire *de novo* oncogenic potential.

The solute carrier organic anion transporter family member 1B3, encodes organic anion transporting polypeptide 1B3 (*OATP1B3*, or *SLCO1B3*), is a 12-transmembrane transporter with normal expression and function restricted to the liver [131]. Several studies have shown that this gene is ectopically expressed in solid tumors of non-hepatic origin, particularly colon cancer [131–134]. Investigations into the cause of this ectopic expression revealed that the normal liver-restricted promoter is silent in these cancers, with expression of “cancer-type” (Ct)-OATP1B3 being driven from an alternative promoter in the second canonical intron [133, 134]. While not previously reported as being within a TE, we noted that this alternative promoter maps within the 5’ LTR (LTR7) of a partly full-length antisense HERV-H element that is missing the 3’ LTR. Expression of HERV-H itself and LTR7-driven chimeric long non-coding RNAs is a noted feature of embryonic stem cells and normal early embryogenesis, where several studies indicate an intriguing role for this ERV group in pluripotency (for recent reviews see [8, 10, 60]). A few studies have also noted higher general levels of HERV-H transcription in colon cancer [109, 135]. The LTR7-driven isoform of *SLCO1B3* makes a truncated protein lacking the first 28 amino acids but also includes protein sequence from the LTR7 and an adjacent MER4C LTR (Fig. 1f). The novel protein is believed to be intracellular and its role in cancer remains unclear. However, one study showed that high expression of this isoform is correlated with reduced progression-free survival in colon cancer [136].

In another study designed specifically to look for TE-initiated chimeric transcripts, we screened RNA-seq libraries from 101 patients with diffuse large B-cell lymphoma (DLBCL) of different subtypes [137] and compared to transcriptomes from normal B-cells. This screen resulted in the detection of 98 such transcripts that were found in at least two DLBCL cases and no normals [138]. One of these involved the gene for fatty acid binding protein 7 (*FABP7*). FABP7, normally expressed in brain, is a member of the FABP family of lipid chaperones involved in fatty acid uptake and trafficking [139]. Overexpression of FABP7 has been reported in several solid tumor types and is associated with poorer prognosis in aggressive breast cancer [139, 140]. In 5% of the DLBCL cases screened, we found that *FABP7* is expressed from an antisense LTR2 (the 5’LTR of a HERV-E element) (Fig. 1g). Since the canonical ATG is in the first exon of *FABP7*, the LTR driven transcript encodes a chimeric protein with a different N-terminus (see accession NM_001319042.1) [138]. Functional analysis in DLBCL cell lines revealed that the LTR-FABP7 protein isoform is required for optimal cell growth and also has subcellular localization properties distinct from the native form [138].

Overall, among all TE types giving rise to chimeric transcripts detected in DLBCL, LTRs were over represented compared to their genomic abundance and, among LTR groups, we found that LTR2 elements and THE1 LTRs were over represented [138]. As discussed above, this predominance of LTRs over other TE types is expected.

### TE-initiated non-coding RNAs in cancer

Since TEs, particularly ERV LTRs, provide a major class of promoters for long non-coding RNAs [56, 141, 142], it is not surprising that multiple LTR-driven lncRNAs have been shown to be involved in cancer. These cases can be broadly divided into those with direct, measurable oncogenic properties (Table 3) and those with expression correlated with a cancer. It should be noted that we have likely missed some examples if the nature of the promoter was not highlighted or mentioned in the original publications. Unlike the coding genes discussed above which have non-TE or native promoters in normal tissues, the lncRNAs described here typically have LTRs as their only promoter in normal or malignant cells.

**Table 3:** LTR-driven LncRNAs with oncogenic role

lncRNA^a^	TE type	TE promoter coordinates (hg38)	Cancer type	references
*SchLAP1*	(ERV1) LTR12C	chr2:180691205–180692425	prostate	[143]
*ROR*	(ERV1) LTR7	chr18:57072052–57072502	breast, others	[147, 150]
*HOST2*	(ERV1) LTR2B	chr10:84171987–84172465	ovarian	[154]
*AFAP1-AS1*	(ERVL-MaLR) THE1A LTR^b^	chr4:7753884–7754236	several	[156, 158]
*SAMMSON*	(ERV1) LTR1A2^b^	chr3:69999501–70000359	melanoma	[161]
*HULC*	(ERVL-MaLR) MLT1A LTR	chr6:8652095–8652454	liver	[163]
*UCA1*	(ERV1) LTR7C	chr19:15828738–15829200	several	[165, 167]
*BANCR*	(ERV1) MER41B LTR	chr9:69306939–69307567	melanoma, others	[169]

^a^Only those cases with supporting evidence of a role in the cancer are listed

^b^The fact that the promoter is an LTR was not previously noted

#### TE-initiated LncRNAs with oncogenic properties

**Fig. 3:**
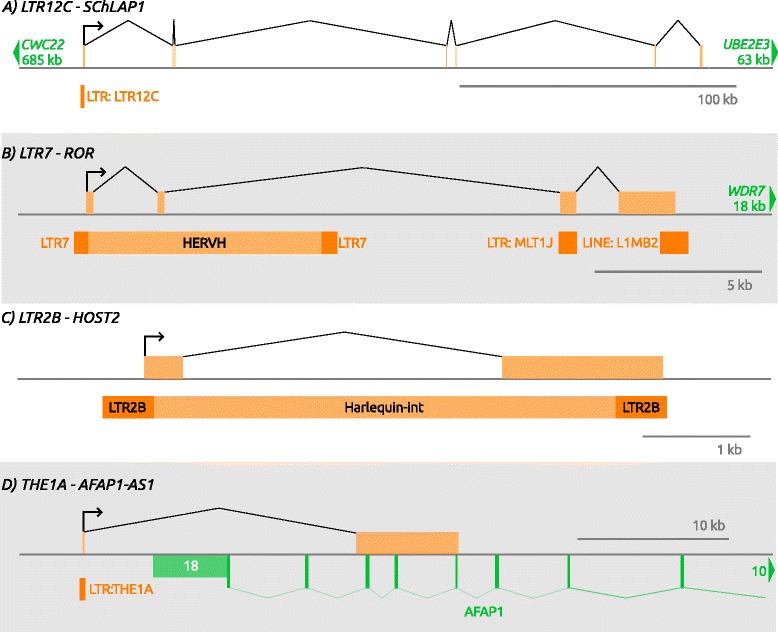
Gene models of select lncRNAs initiating within LTRs that are involved in oncogenesis. **a** A solitary LTR12C element initiates *SChLAP1,* a long inter-genic non-coding RNA. **b** The 5’ LTR7 of a full-length HERVH element initiates the lncRNA *ROR,* with an exon partially incorporating internal ERV sequence. **c** The *HOST2* lncRNA is completely derived from components of a Harlequin (or HERV-E) endogenous retrovirus and its flanking LTR2B. **d** Anti-sense to the *AFAP1* gene, a THE1A LTR initiates transcription of the lncRNA *AFAP1-AS1.* The second exon of *AFAP1-AS1* overlaps exons 14–16 of *AFAP1*, possibly leading to RNA interference of the gene

In an extensive study, Prensner et al. reported that the lncRNA *SchLAP1* (SWI/SNF complex antagonist associated with prostate cancer 1) is overexpressed in ~25% of prostate cancers, is an independent predictor of poor clinical outcomes and is critical for invasiveness and metastasis [143]. Intriguingly, they found that *SchLAP1* inhibits the function of the SWI/SNF complex, which is known to have a tumor suppressor roles [144]. While not mentioned in the main text, the authors report in supplementary data that the promoter for this lncRNA is an LTR (Fig. 3a). Indeed, this LTR is a sense-oriented solitary LTR12C (of the ERV9 group).

*Linc-ROR* is a non-coding RNA (long intergenic non-protein coding RNA, regulator of reprogramming) promoted by the 5’ LTR (LTR7) of a full length HERV-H element [56] (Fig. 3b) and has been shown to play a role in human pluripotency [145]. Evidence suggests it acts as a microRNA sponge of miR-145, which is a repressor of the core pluripotency transcription factors Oct4, Nanog and Sox2 [146]. Several recent studies have reported an oncogenic role for *Linc-ROR* in different cancers by sponging miR-145 [147–149] or through other mechanisms [150, 151].

Using Serial Analysis of Gene Expression (SAGE), Rangel et al. identified five Human Ovarian cancer Specific Transcripts (HOSTs) that were expressed in ovarian cancer but not in other normal cells or cancer types examined [152]. One of these, *HOST2*, is annotated as a spliced lncRNA entirely contained within a full length HERV-E and promoted by an LTR2B element (Fig. 3c). Perusal of RNA-Seq from the 9 core ENCODE cell lines shows robust expression of *HOST2* in GM12878, a B-lymphoblastoid cell line, which extends beyond the HERV-E. As with *Linc-ROR*, *HOST2* appears to play an oncogenic role by functioning as a miRNA sponge of miRNA *let-7b,* an established tumor suppressor [153], in epithelial ovarian cancer [154].

The Ref-Seq annotated lncRNA *AFAP1* antisense RNA 1 (*AFAP1-AS1*) runs antisense to the actin filament associated protein 1 (*AFAP1*) gene and several publications report its up-regulation and association with poor survival in a number of solid tumor types [155–158]. While the oncogenic mechanism of *AFAP1-AS1* has not been extensively studied, one report presented evidence that it promotes cell proliferation by upregulating RhoA/Rac2 signaling [159] and its expression inversely correlates with *AFAP1*. Although clearly annotated as initiating within a solitary THE1A LTR (Fig. 3d), this fact has not been mentioned in previous publications. In screens for TE-initiated transcripts using RNA-seq data from HL cell lines, we noted recurrent and cancer-specific up-regulation of *AFAP1-AS1* (unpublished observations), suggesting that it is not restricted to solid tumors. The inverse correlation of expression between *AFAP1* and *AFAP1-AS1* suggests an interesting potential mechanism by which TE-initiated transcription may suppress a gene; where an anti-sense TE-initiated transcript disrupts the transcription, translation or stability of a tumor suppressor gene transcript through RNA interference [160].

The *SAMMSON* lncRNA (survival associated mitochondrial melanoma specific oncogenic non-coding RNA), which is promoted by a solitary LTR1A2 element, was recently reported as playing an oncogenic role in melanoma [161]. This lncRNA is located near the melanoma-specific oncogene *MITF* and is always included in genomic amplifications involving *MITF*. Even in melanomas with no genomic amplification of this locus, *SAMMSON* is expressed in most cases, increases growth and invasiveness and is a target for SOX10 [161], a key TF in melanocyte development which is deregulated in melanoma [162]. Interestingly, the two SOX10 binding sites near the SAMMSON TSS lie just upstream and downstream of the LTR (Fig. 2b), suggesting that both the core promoter motifs provided by the LTR and adjacent enhancer sites combine to regulate *SAMMSON*.

Other examples of LTR-promoted oncogenic lncRNAs include *HULC* for Highly Upregulated in Liver Cancer [163, 164], *UCA1* (urothelial cancer associated 1) [165–168] and *BANCR* (BRAF-regulated lncRNA 1) [169–171]. Although not mentioned in the original paper, three of the four exons of *BANCR* were shown to be derived from a partly full length MER41 ERV, with the promoter within the 5’LTR of this element annotated MER41B [141]. Intriguingly, MER41 LTRs were recently shown to harbor enhancers responsive to interferon, indicating a role for this ERV group in shaping the innate immune response in primates [52]. It would be interesting to investigate roles for *BANCR* with this in mind.

#### TE-initiated lncRNAs as cancer-specific markers

There are many examples of TE-initiated RNAs with potential roles in cancer or which are preferentially expressed in malignant cells but for which a direct oncogenic function has not yet been demonstrated. Still, such transcripts may underlie a predisposition for transcription of specific groups of LTRs/TEs in particular malignancies and therefore function as a marker for a cancer or cancer subtype. Since these events potentially do not confer a fitness advantage for the cancer cell, they are not “exaptations” but “nonaptations” [4].

One of these is a very long RNA initiated by the antisense promoter of an L1PA2 element as reported by Tufarelli’s group and termed *LCT13* [172, 173]. EST evidence indicates splicing from the L1 promoter to the *GNTG1* gene, located over 300 kb away. The tumor suppressor gene, tissue factor pathway inhibitor 2, (*TFPI-2)*, which is often epigenetically silenced in cancers [174], is antisense to LCT13 and it was shown that LCT13 transcript levels are correlated with down regulation of *TFPI-2* and associated with repressive chromatin marks at the *TFPI-2* promoter [172].

Gibb et al. analyzed RNA-Seq from colon cancers and matched normal colon to find cancer-associated lncRNAs and identified an RNA promoted by a solitary MER48 LTR, which they termed *EVADR*, for Endogenous retroviral-associated ADenocarcinoma RNA [175]. Screening of data from The Cancer Genome Atlas (TCGA) [176] showed that *EVADR* is highly expressed in several types of adenocarcinomas, it is not associated with global activation of MER48 LTRs across the genome and its expression correlated with poorer survival [175]. In another study, Gosenca et al. used a custom microarray to measure overall expression of several HERV groups in urothelial carcinoma compared to normal urothelial tissue and generally found no difference [111]. However, they found one full-length HERV-E element, located in the antisense direction in an intron of the *PLA2G4A* gene that is transcribed in urothelial carcinoma and appears to modulate *PLA2G4A* expression, thereby possibly contributing to carcinogenesis, although the mechanism is not clear.

By mining long nuclear RNA datasets from ENCODE cell lines, normal blood and Ewing sarcomas, one group identified over 2000 very long (~50–700 kb) non coding transcripts termed vlincRNAs [142]. They found the promoters for these vlincRNAs to be enriched in LTRs, particularly for cell type-specific vlincRNAs, and the most common transcribed LTR types varied in different cell types. Moreover, among the datasets examined, they reported that the number of LTR-promoted vlincRNAs correlated with degree of malignant transformation, prompting the conclusion that LTR-controlled vlincRNAs are a “hallmark” of cancer [142].

In a genome-wide CAGE analysis of 50 hepatocellular carcinoma (HCC) primary samples and matched non-tumor tissue, Hashimoto et al. found that many LTR-promoted transcripts are upregulated in HCC, most of these apparently associated with non-coding RNAs as the CAGE peaks in the LTRs are far from annotated protein coding genes [177]. Similar results were found in mouse HCC. Among the hundreds of human LTR groups, they found the LTR-associated CAGE peaks to be significantly enriched in LTR12C (HERV9) LTRs and mapped the common TSS site within these elements, which agrees with older studies on TSS mapping of this ERV group [178]. Moreover, this group reported that HCCs with highest LTR activity mostly had a viral (Hepatitis B) etiology, were less differentiated and had higher risk of recurrence [177]. This study suggests widespread tissue-inappropriate transcriptional activity of LTRs in HCC.

#### LTR12s as flexible promoters in cancer and normal tissues

Most recent human ERV LTR research has been focused on HERV-H (LTR7/7Y/7B/7C) due to roles for HERV-H/LTR7-driven RNAs in pluripotency [56–58, 60, 179, 180] or on the youngest HERV group, HERV-K (LTR5/5Hs), due to its expression in early embryogenesis [181–183], coding capacity of some members [30, 184] and potential roles for its proteins in cancer and other diseases [30–33, 185]. LTR12s (including LTR12B,C,D,E and F subtypes), which are the LTRs associated with the HERV-9 group [186], are generally of similar age to HERV-H [187] but are much more numerous than HERV-H or HERV-K, with solitary LTRs numbering over 6000 (Table 1). There are several examples of LTR12s providing promoters for coding genes or lncRNAs in various normal tissues [63, 188–191]. LTR12s, particularly LTR12C, are longer and more CpG rich than most other ERV LTRs, possibly facilitating development of diverse inherent tissue-specificities and flexible combinations of TF binding sites, which may be less probable for other LTR types. For example, the consensus LTR7 (HERV-H) is 450 bp whereas LTR12C (of similar age) is 1577 bp [114], which is usually long for retroviral LTRs. As noted above, LTR12 elements are among the most enriched LTR types activated as promoters in HCC [177] and appear to be the most active LTR type in K562 cells [142]. It is important to point out, however, that only a very small fraction of genomic LTR12 copies are transcriptionally active in any of these contexts, so general conclusions about activity of ‘a family of LTRs’ should be made with caution.

A number of other recent investigations on LTR12-driven chimeric transcription have been published. One study specifically screened for and detected numerous LTR12-initiated transcripts in ENCODE cell lines, some of which extend over long genomic regions and emanate from bidirectional promoters within these LTRs [192]. The group of Dobbelstein discovered that a male germ line-specific form of the tumor suppressor *TP63* gene is driven by an LTR12C [190]. Interestingly, they found that this LTR is silenced in testicular cancer but reactivated upon treatment with histone deacetylase inhibitors (HDACi), which also induces apoptosis [190]. In follow-up studies, this group used 3’ RACE to detect more genes controlled by LTR12s in primary human testis and in the GH testicular cancer cell line and reported hundreds of transcripts, including an isoform of *TNFRSF10B* which encodes the death receptor DR5 [193]. As with *TP63*, treating GH or other cancer cell lines with HDAC inhibitors such as trichostatin A activated expression of the LTR12-driven *TNFRSF10B* and some other LTR12-chimeric transcripts and induced apoptosis [193, 194]. Therefore, in some cases, LTR-driven genes can have a proapoptotic role. In accord with this notion is a study reporting that LTR12 antisense U3 RNAs were expressed at higher levels in non-malignant versus malignant cells [195]. It was proposed that the antisense U3 RNA may act as a trap for the transcription factor NF-Y, known to bind LTR12s [196], and hence participate in cell cycle arrest [195].

### Chromosomal translocations involving TEs in cancer

Activation or creation of oncogenes via chromosomal translocations most commonly involves either the fusion of two coding genes or juxtaposition of new regulatory sequences next to a gene, resulting in oncogenic effects due to ectopic expression [197]. One might expect some of the latter cases to involve TE-derived promoters/enhancers but, to date, there are very few well-documented examples of this mechanism in oncogenesis. The ETS family member *ETV1* (ETS variant 1) is a transcription factor frequently involved in oncogenic translocations, particularly in prostate cancer [198]. Although not a common translocation, Tomlins et al. identified a prostate tumor with the 5’ end of a HERV-K (HML-2) element on chromosome 22q11.23 fused to *ETV1* [199]. This particular HERV-K element is a complex locus with two 5’ LTRs and is quite highly expressed in prostate cancer [200]. Indeed, while a possible function is unknown, this HERV-K locus produces a lncRNA annotated as PCAT-14, for prostate cancer–associated ncRNA transcript-14 [201]. In the HERV-K-ETV1 fusion case, the resultant transcript (Genbank Accession EF632111) initiates in the upstream 5’LTR, providing evidence that the LTR controls expression of *ETV1*.

The fibroblast growth factor receptor 1 *(FGFR1)* gene on chromosome 8 is involved in translocations with at least 14 partner genes in stem cell myeloproliferative disorder and other myeloid and lymphoid cancers [202]. One of these involves a HERVK3 element on chromosome 19 and this event creates a chimeric ORF with HERVK3 gag sequences [203]. While it was reported that the LTR promoter may contribute to expression of the fusion gene [203], no supporting evidence was presented. Indeed, perusal of public expression data (Expressed sequence tags) from a variety of tissues indicates that the HERVK3 element on chromosome 19 is highly expressed, but from a non-ERV promoter just upstream (see chr19:58,305,253–58,315,303 in human hg38 assembly). Therefore, there is little current evidence for LTR/TE promoters playing a role in oncogene activation via chromosomal translocations or rearrangements.

### Models for onco-exaptation

The aforementioned cases of onco-exaptation are a distinct mechanism by which proto-oncogenes become oncogenic. Classical activating mutations within TEs may also lead to transcription of downstream oncogenes but we are unaware of any evidence for DNA mutations resulting in LTR/TE transcriptional activation, including cases where local DNA was sequenced [38] (unpublished results). Thus, it is important to consider the etiology through which LTRs/TEs become incorporated into new regulatory units in cancer. The mechanism could possibly be therapeutically or diagnostically important and perhaps even model how TEs influence genome regulation in evolutionary time.

In some of the above examples, there is no or very little detectable transcription from the LTR/TE in any cell type other than the cancer type in which it was reported, suggesting the activity is specific to a particular TE in a particular cancer. In other cases, CAGE or EST data show that the LTR/TE can be expressed in other normal or cancer cell types, perhaps to a lower degree. Hence the term “cancer-specific” should be considered a relative one. Indeed, the idea that the same TE-promoted gene transcripts occur recurrently in tumors from independent individuals is central to understanding how these transcripts arise. Below we present two models that may explain the phenomenon of onco-exaptation.

#### The De-repression model

**Fig. 4:**
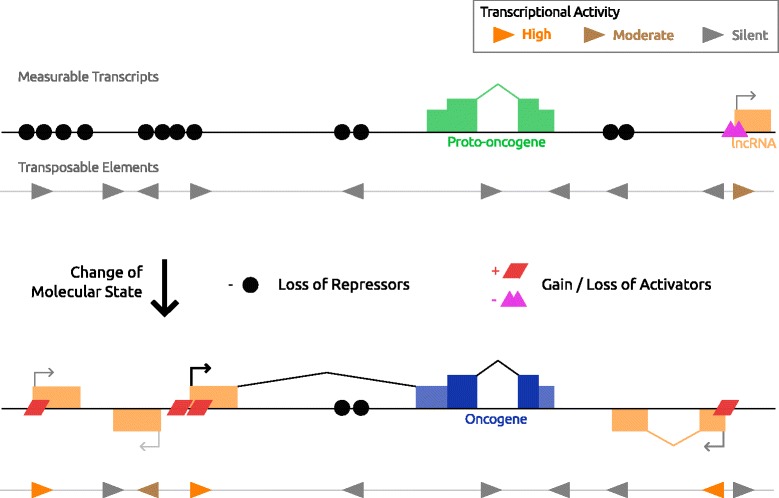
De-repression model for onco-exaptation. In the normal or pre-malignant state TEs (grey triangles) are largely silenced across the genome. There is low transcriptional activity to produce long non-coding RNA (orange box), or express coding genes in the case of evolutionary exaptations (not shown). The example proto-oncogene (green box) is under the regulatory control of its native, restrictive promoter. During the process of transformation and/or oncogenesis, a change in the molecular state of the cell occurs leading to loss of TE repressors (black circles), i.e. DNA hypomethylation, loss of transcriptional or epigenetic repressive factors. The change could also be accompanied by a change/gain in activating factor activities (red and purple shapes). Together these de-repression events result in higher TE promoter activity (orange triangles) and more TE-derived transcripts based on the factors that become deregulated. Oncogenic activation of proto-oncogenes is a consequence of a particular molecular milieu that arises in the cancerous cells

Lamprecht and co-workers proposed a ‘De-repression model’ for the LTR driven transcription of *CSF1R* [204]. The distinguishing feature of this model is that onco-exaptations arise deterministically, as a consequence of molecular changes that occur during oncogenesis, changes which act to de-repress LTRs or other TEs (Fig. 4). It follows that ‘activation’ of normally dormant TEs/LTRs could lead to robust oncogene expression. In the *CSF1R* case, the *THE1B* LTR, which promotes *CSF1R* in HL, contains binding sites for the transcription factors Sp1, AP-1 and NF-kB, each of which contributes to promoter activity in a luciferase reporter experiment [113]. High NF-kB activity, which is known to be up-regulated in HL, loss of the epigenetic corepressor CBFA2T3 as well as LTR hypomethylation all correlated with CSF1R-positive HL driven by the LTR [113]. Under the de-repression model, the *THE1B* LTR is repressed by default in the cell but under a particular set of conditions (gain of NF-kB, loss of CBFA2T3, loss of DNA methylation) the LTR promoter is remodeled into an active state [204]. More generally, the model proposes that a particular LTR activation is a consequence of the pathogenic or disrupted molecular state of the cancer cell. In a similar vein, Weber et al. proposed that the L1-driven transcription of *MET* arose as a consequence of global DNA hypomethylation and loss of repression of TEs in cancer [124].

The *LOR1a*-*IRF5* onco-exaptation in HL [117] can be interpreted using a de-repression model. An interferon regulatory factor binding element site was created at the intersection of the *LOR1a* LTR and genomic DNA. In normal and HL cells negative for *LOR1a-IRF5*, the LTR is methylated and protected from DNAse digestion, a state that is lost in de-repressed HL cells. This transcription factor-binding motif is responsive to IRF5 itself and creates a positive feedback loop between the IRF5 and the chimeric *LOR1a-IRF5* transcript. Thus epigenetic de-repression of this element may reveal an oncogenic exploitation, resulting in high recurrence of *LOR1a* LTR-driven IRF5 in HL [117].

A de-repression model explains several experimental observations, such as the necessity for a given set of factors to be present (or absent) for a certain promoter to be active, especially when those factors differ between cell states. Indeed, experiments probing the mechanism of TE/LTR activation have used this line of reasoning, often focusing on DNA methylation [113, 117, 125, 129]. The limitation of these studies is that they fail to determine if a given condition is sufficient for onco-exaptation to arise. For instance, the human genome contains >37,000 *THE1* LTR loci (Table 1), and indeed this set of LTRs is generally more active in HL cells compared to B-cells as would be predicted [113] (unpublished results). The critical question is why particular *THE1* LTR loci, such as *THE1B-CSF1R*, are recurrently de-repressed in HL, yet thousands of homologous LTRs are not.

#### The Epigenetic Evolution model

**Fig. 5:**
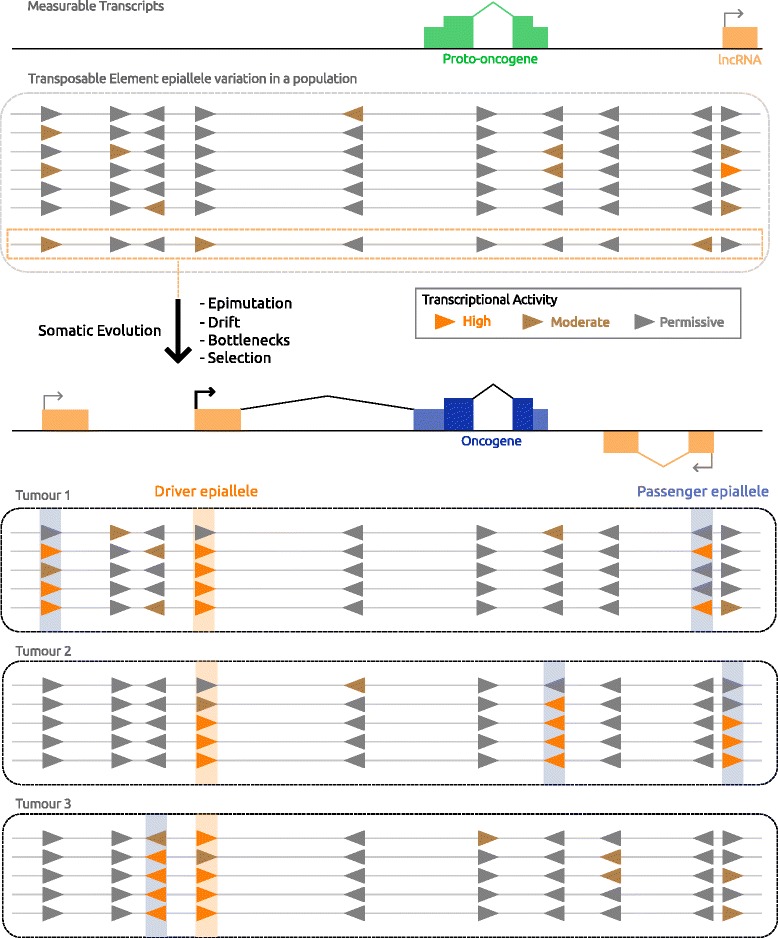
Epigenetic evolution model for onco-exaptation. In the starting cell population there is a dispersed and low/noisy promoter activity at TEs (colored triangles) from a set of transcriptionally permissive TEs (grey triangles). TE-derived transcript expression is low and variable between cells. Some transcripts are more reliably measurable (orange box). Clonal tumor evolutionary forces change the frequency and expression of TE-derived transcripts by homogenizing epialleles and use of TE promoters (highlighted haplotype). A higher frequency of ‘active’ TE epialleles at a locus results in increased measurable transcripts initiating from that position. TE epialleles that promote oncogenesis, namely onco-exaptations, can be selected for and arise multiple times independently as driver epialleles, in contrast to the more dispersed passenger epialleles, or “nonaptations”

A central premise in the TE field states that TEs can be beneficial to a host genome since they increase genetic variation in a population and thus increase the rate at which evolution (by natural selection) occurs [62, 205, 206]. The epigenetic evolution model for onco-exaptation (Fig. 5) draws a parallel to this premise within the context of tumor evolution.

Key to the epigenetic evolution model is that there is high epigenetic variance, both between LTR loci and at the same LTR locus between cells in a population. This epigenetic variance fosters regulatory innovation, and increases during oncogenesis. In accord with this idea are several studies showing that DNA methylation variation, or heterogeneity, increases in tumor cell populations and this isn’t simply a global hypomethylation relative to normal cells [207–209] (reviewed in [210]). In contrast to the de-repression model, a particular pathogenic molecular state is not sufficient or necessary for TE-driven transcripts to arise; instead the given state only dictates which sets of TEs in the genome are permissive for transcription. Likewise, global de-repression events, such as DNA hypomethylation or mutation of epigenetic regulators, are not necessary, but would increase the rate at which novel transcriptional regulation evolves.

Underpinning this model is the idea that LTRs are highly abundant and self-contained promoters dispersed across the genome that can stochastically initiate low or noisy transcription. This transcriptional noise is a kind of epigenetic variation and thus contributes to cell-cell variation in a population. Indeed, by re-analyzing CAGE datasets of retrotransposon-derived TSSs published by Faulkner et al. [73], we observed that TE-derived TSSs have lower expression levels and are less reproducible between biological replicates, compared to non-TE promoters (unpublished observations). During malignant transformation, TFs can become deregulated and genome-wide epigenetic perturbations occur [94, 98, 211] which would change the set of LTRs that are potentially active as well as possibly increasing the total level of LTR-driven transcriptional noise. Up-regulation of specific LTR-driven transcripts would initially be weak and stochastic, from the set of permissive LTRs. Those cells gaining an LTR-driven transcript which confers a growth advantage would then be selected for, and the resultant oncogene expression would increase in the tumor population as that epiallele increases in frequency, in a similar fashion as proposed for the epigenetic silencing of tumor suppressor genes [95, 99, 100]. Notably, this scenario also means that within a tumor, LTR-driven transcription would be subject to epigenetic bottleneck effects as well, and that transcriptional LTR noise can become “passenger” expression signals as the cancer cells undergo somatic, clonal evolution.

It may be counter-intuitive to think of evolution and selection as occurring outside the context of genetic variation, but the fact that both genetic mutations and non-genetic/epigenetic variants can contribute to somatic evolution of a cancer is becoming clear [209, 212–215]. Epigenetic information or variation by definition is transmitted from mother to daughter cells. Thus, in the specific context of a somatic/asexual cell population such as a tumor, this information, which is both variable between cells in the population and heritable, will be subject to evolutionary changes in frequency. DNA methylation in particular has a well-established mechanism by which information (mainly gene repression) is transmitted epigenetically from mother to daughter cells [216] and DNA hypomethylation at LTRs often correlates with their expression [113, 117, 217]. Thus, this model suggests that one important type of “epigenetic variant” or epiallele is the transcriptional status of the LTR itself, since the phenotypic impact of LTR transcription may be high in onco-exaptation. Especially in light of the fact that large numbers of these highly homologous sequences are spread across the genome, epigenetic variation, and possibly selection, at LTRs creates a fascinating system by which epigenetic evolution in cancer may occur.

## Conclusions

Here we have reviewed the growing number of examples of LTR/TE onco-exaptation. Although such TEs have the potential to be deleterious by contributing to oncogenesis if transcriptionally activated, their fixation in the genome and ancient origin suggests that their presence is not subject to significant negative selection. This could be due to the low frequency of onco-exaptation at a particular TE locus and/or to the fact that cancer is generally a disease that occurs after the reproductive years. However, it is generally assumed that negative selection is the reason why TEs are underrepresented near or within genes encoding developmental regulators [218–220]. Similarly we hypothesize that LTR/TE insertions predisposed to causing potent onco-exaptations at a high frequency would also be depleted by selective forces.

In this review we have also presented two models that may explain such onco-exaptation events. These two models are not mutually exclusive but they do provide alternative hypotheses by which TE-driven transcription may be interpreted. This dichotomy is possibly best exemplified by the *ERBB4* case (Fig. 1e) [129]. There are two LTR-derived promoters which result in aberrant *ERBB4* expression in ALCL. From the de-repression model viewpoint, both LTR elements are grouped MLT1 (MLT1C and MLT1H) and thus this group can be interpreted as de-repressed. From the epigenetic evolution model viewpoint, this is convergent evolution/selection for onco-exaptations involving *ERBB4*.

Through application of the de-repression model, TE-derived transcripts could be used as a diagnostic marker in cancer. If the set of TE/LTR derived transcripts are a deterministic consequence of a given molecular state, by understanding which set of TEs correspond to which molecular state, it might be possible to assay cancer samples for functional molecular phenotypes. In HL for example, CSF1R status is prognostically important [115] and this is dependent on the transcriptional state of a single *THE1B.* HL also has a specific increase in *THE1* LTR transcription genome-wide (unpublished observations). Thus, it’s reasonable to hypothesize that the prognostic power can be increased if the transcriptional status of all THE1 LTRs is considered. A set of LTRs can then be interpreted as an in situ ‘molecular sensor’ for aberrant NF-kB function in HL/B-cells for instance.

The epigenetic evolution model proposes that LTR-driven transcripts can be interpreted as a set of epimutations in cancer, similar to how oncogenic mutations are analyzed. Genes that are recurrently (and independently) onco-exapted in multiple different tumors of the same cancer type may be a mark of selective pressure for acquiring that transcript. This is distinct from the more diverse/noisy “passenger LTR” transcription occurring across the genome. These active but “passenger LTRs” may be expressed to a high level within a single tumor population due to epigenetic drift and population bottlenecks but would be more variable across different tumors. Thus analysis of recurrent and cancer-specific TE-derived transcripts may enrich for genes of significance to tumor biology.

While we focused in this review on TE-initiated transcription in cancer, many of the concepts presented here can be applied to other regulatory functions of TEs such as enhancers, insulators, or repressors of transcription. Although less straightforward to measure, it is probable that perturbations to such TE regulatory functions contribute to some malignancies. Furthermore, several studies have shown that TEs play substantial roles in cryptic splicing in humans [221–223] and thus may be a further substrate of transcriptional innovation in cancer, particularly since DNA methylation state can affect splicing [224].

Regardless of the underlying mechanism, onco-exaptation offers a tantalizing opportunity to model evolutionary exaptation. Specifically, questions such as “How do TEs influence the rate of transcriptional/regulatory change?” can be tested in cell culture experiments. As more studies that focus on regulatory aberrations in cancer are performed in the coming years, we predict that this phenomenon will become increasingly recognized as a significant force shaping transcriptional innovation in cancer. Moreover, we propose that studying such events will provide insight into how TEs have contributed to reshaping transcriptional patterns during species evolution.

## Abbreviations

AFAP1-AS1: AFAP1 antisense RNA 1
ALCL: Anaplastic large-cell lymphoma
ALK: Anaplastic lymphoma kinase
BANCR: BRAF-regulated lncRNA 1
CAGE: Capped analysis of gene expression
CSFIR: Colony stimulating factor one receptor
DLBCL: Diffuse large B-cell lymphoma
ERBB4: Erb-b2 receptor tyrosine kinase 4
ERV: Endogenous retrovirus
EST: Expressed sequence tag
ETV1: ETS variant 1
EVADR: Endogenous retroviral-associated Adenocarcinoma RNA
FABP7: Fatty acid binding protein 7
HCC: Hepatocellular carcinoma
HL: Hodgkin lymphoma
HOST2: Human ovarian cancer specific transcript-2
HULC: Highly upregulated in liver cancer
IRF5: Interferon regulatory Factor 5
IRFE: Interferon regulatory factor-binding element
Linc-ROR: Long intergenic non-protein coding RNA, regulator of reprogramming
LINE-1: L1: Long interspersed repeat-1
LncRNA: Long non-coding RNA
LTR: Long terminal repeat
MET: MET proto-oncogene, receptor tyrosine kinase
OAT1B3: Organic anion transporting polypeptide 1B3
SAMMSON: Survival associated mitochondrial melanoma specific oncogenic non-coding RNA
SchLAP1: SWI/SNF complex antagonist associated with prostate cancer 1
SINE: Short interspersed element
SLCO1B3: Solute carrier organic anion transporter family member 1B3
TCGA: The cancer genome atlas
TE: Transposable element
TF: Transcription factor
TFPI-2: Tissue factor pathway inhibitor 2
TIS: Translation initiation site
TSS: Transcriptional start site
UCA1: Urothelial cancer associated 1.
